# Analysis of Longitudinal Studies With Repeated Outcome Measures: Adjusting for Time-Dependent Confounding Using Conventional Methods

**DOI:** 10.1093/aje/kwx311

**Published:** 2017-09-06

**Authors:** Ruth H Keogh, Rhian M Daniel, Tyler J VanderWeele, Stijn Vansteelandt

**Affiliations:** 1 Department of Medical Statistics, London School of Hygiene and Tropical Medicine, London, United Kingdom; 2 Division of Population Medicine, Cardiff University, Cardiff, United Kingdom; 3 Department of Epidemiology, Harvard T.H. Chan School of Public Health, Boston, Massachusetts; 4 Department of Biostatistics, Harvard School of Public Health, Boston, Massachusetts; 5 Department of Applied Mathematics and Computer Science, Ghent University, Ghent, Belgium

**Keywords:** direct effect, indirect effect, inverse probability weight, longitudinal study, marginal structural model, sequential conditional mean model, time-varying confounder, total effect

## Abstract

Estimation of causal effects of time-varying exposures using longitudinal data is a common problem in epidemiology. When there are time-varying confounders, which may include past outcomes, affected by prior exposure, standard regression methods can lead to bias. Methods such as inverse probability weighted estimation of marginal structural models have been developed to address this problem. However, in this paper we show how standard regression methods can be used, even in the presence of time-dependent confounding, to estimate the total effect of an exposure on a subsequent outcome by controlling appropriately for prior exposures, outcomes, and time-varying covariates. We refer to the resulting estimation approach as sequential conditional mean models (SCMMs), which can be fitted using generalized estimating equations. We outline this approach and describe how including propensity score adjustment is advantageous. We compare the causal effects being estimated using SCMMs and marginal structural models, and we compare the two approaches using simulations. SCMMs enable more precise inferences, with greater robustness against model misspecification via propensity score adjustment, and easily accommodate continuous exposures and interactions. A new test for direct effects of past exposures on a subsequent outcome is described.

This paper discusses estimation of causal effects from studies with longitudinal repeated measures of exposures and outcomes, such as when individuals are observed at repeated visits. Interest may lie in studying the “total effect” of an exposure at a given time on a concurrent or subsequent outcome or in the effect of a pattern of exposures over time on a subsequent outcome. These different types of effects are defined below. Special methods have been developed to handle the complications of the time-dependent confounding that can occur in this longitudinal setting (1), inverse probability weighted (IPW) estimation of marginal structural models (MSMs) being the most commonly employed, as well as others including g-computation and g-estimation. Good introductions to these methods are available (2, 3), and while the other g-methods are still not widely used, IPW estimation of MSMs is becoming more commonplace. In this paper we show how, in fact, conventional methods can be used to estimate “total effects,” even in the presence of time-dependent confounding, by controlling for prior exposures, outcomes, and time-varying covariates. That is, we provide a reminder that it is not always necessary to default to using IPW estimation of MSMs or g-methods when there are time-varying confounders. While standard regression adjustment is often employed in studies using longitudinal measures, issues of potential biases due to time-dependent confounding are not always carefully considered and do indeed result in bias if prior values of the exposure and outcome are not controlled for.

The methods described in this paper are based on sequential conditional mean models (SCMMs) for the repeated outcome measures, fitted using generalized estimating equations (GEEs). We set out the important considerations for securing results against bias due to model misspecification and compare the effects that can be estimated using SCMMs and IPW estimation of MSMs, as well as comparing the methods in simulation studies. IPW estimation of MSMs uses weighted regressions in which each individual’s data at each time point receives a weight equal to the inverse of an estimated probability that that person had their observed exposures until that time, given their other covariates up to that time. A drawback is that some individuals may have a large weight, which causes finite-sample bias and imprecision, even when using stabilized weights. This occurs particularly in studies with many visits or continuous exposures (4, 5). Several applications using IPW estimation of MSMs have in fact considered total, particularly short-term, effects (6–8) where simpler methods may have been suitable and more efficient.

We also present a new test of whether there are direct effects of past exposures on a subsequent outcome not mediated through intermediate exposures. The test can be used in conjunction with the conventional methods as part of an analysis strategy to inform whether more complex analyses are needed to estimate certain effects.

## ESTIMATING TOTAL EXPOSURE EFFECTS

### Setup and notation

**Figure 1.**
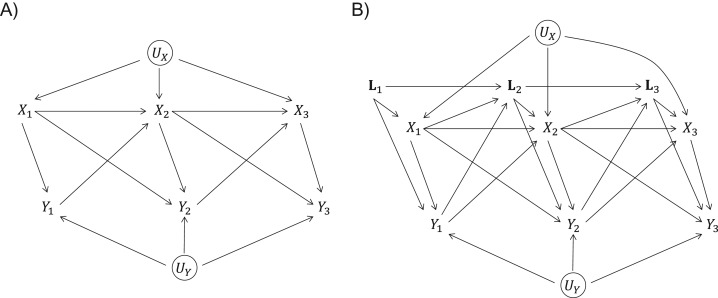
Associations between an exposure $X_{t}$ and outcome $Y_{t}$ measured longitudinally, with random effects $U_{X}$ and $U_{Y}$ (circles indicate that these are unobserved). A) Without time-varying confounders. B) With time-varying confounders.

Individuals are observed at T visits, t=1,…,T, at which we observe the outcome $Y_{t}$, the exposure $X_{t}$, and a vector of covariates $L_{t}$. Figure 1 depicts how variables may be related over time. $U_{Y}$ and $U_{X}$ denote unobserved random effects affecting $Y_{t}$ and $X_{t}$ respectively. The set of measures up to time t is indicated using a bar (e.g., ${\bar{X}}_{t}=(X_{1},\ldots ,X_{t-1},X_{t})$). It is assumed that $X_{t}$ refers to a measure at a time point just before that to which $Y_{t}$ refers. This would occur if $X_{t}$ referred to a status during [t−1,t) and $Y_{t}$ referred to a status during [t,t+1). Sensitivity analyses can be used to investigate assumptions about temporal ordering. We focus on binary exposures and continuous outcomes. Other types of exposures and outcomes are discussed later.

### Defining a total exposure effect

Figure 1 visualizes the primary issues arising in a longitudinal observational setting, notably that prior exposure affects future outcome, prior outcome affects future exposure and covariates, and that there is time-dependent confounding by time-varying covariates $L_{t}$: $L_{t}$ are confounders for the association between $X_{t}$ and $Y_{t}$, but on the pathway from $X_{t-1}$ to $Y_{t}$. Figure 1 could be extended to allow non-time-varying covariates and more lagged effects, (e.g., an arrow from $X_{t-2}$ to $Y_{t}$).

The “total effect” of an exposure at time t−a(a=0,1,…), $X_{t-a}$, on $Y_{t}$ includes both the indirect effect of $X_{t-a}$ on $Y_{t}$ through future exposures $(X_{t-a+1},\ldots ,X_{t})\;$and the direct effect of $X_{t-a}$ on $Y_{t}$ not through future exposures. For example, in Figure 1B the indirect effect of $X_{1}$ on $Y_{2}$ is via the pathways $X_{1}\to X_{2}\to Y_{2}\;$ and $X_{1}\to L_{2}\to X_{2}\to Y_{2}$, and the direct effect is via the pathways $X_{1}\to Y_{2}$ and $X_{1}\to L_{2}\to Y_{2}$. In Figure 1 the total effect of $X_{t}$ on $Y_{t}$ is the same as the direct effect; we also refer to this as the “short-term effect.” In the terminology of mediation, the direct effect corresponds to the “controlled direct effect” (9). We refer to a “long-term direct effect” as the effect of a lagged exposure $X_{t-a}\;(a=0,1,\ldots )$ on a subsequent outcome $Y_{t}$ that is not mediated via intermediate exposures.

This paper does not consider another type of causal effect—the joint effect of a particular pattern of exposures over a series of time points on a subsequent outcome (e.g., the joint effect of $X_{t-1}$ and $X_{t}$ on $Y_{t}$). Our focus is the total effect of a single exposure on a subsequent outcome. Our definition of a total effect does not make any statements about whether a treatment will always be continued once it has started. Such total effects are useful for a doctor making a pragmatic decision about whether to start a patient on a treatment at a given time, accounting for the fact that the patient may subsequently naturally deviate from this treatment (or nontreatment) at a later visit.

To estimate causal effects, we assume no unmeasured confounding. This will generally hold only approximately in an observational setting, and it is hoped that the most important confounders are measured.

### Sequential conditional mean models

We focus first on estimating the short-term effect of $X_{t}$ on $Y_{t}$ (which is also the total effect of $X_{t}$ on $Y_{t}$) and, to discuss the issues arising, first suppose that there is no random effect $U_{Y}$ so that longitudinal outcomes $Y_{t}$ are correlated only via the $X_{t}$ and $L_{t}$. Consider the following model for the expected outcome at time t conditional on exposures and covariates up to time t:

(1) $$E\left(Y_{t}|{\bar{X}}_{t},{\bar{L}}_{t}\right)=\beta_{0}+\beta_{X1}X_{t}+\beta_{X2}X_{t-1}+\beta_{L}^{T}L_{t}.$$

Model (1) is a SCMM. If it is correctly specified and if moreover the history ${\overset{}{X}}_{t-1}$ and ${\overset{}{L}}_{t}$ is sufficient to adjust for confounding of the effect of $X_{t}$ on $Y_{t}$, then parameter $\beta_{X1}$ represents the causal effect of $X_{t}$ on $Y_{t}$. As discussed below, this effect can be estimated by fitting traditional regression models. Interaction terms, variable transformations, terms in and interactions with t, and baseline covariates could be incorporated into the SCMM. Model (1) extends directly to estimation of total effect of $X_{t-a}\;(a=1,2,\ldots )$ on $Y_{t}$, for example:

(2) $$E\left(Y_{t}|{\bar{X}}_{t-a},{\bar{L}}_{t-a}\right)=\beta_{0}+\beta_{X1}X_{t-a}+\beta_{X2}X_{t-a-1}+\beta_{L}^{T}L_{t-a}.$$

In model (2) $\beta_{X1}$ represents the total effect of $X_{t-a}$ on $Y_{t}$.

SCMMs can be used to model total effects. However, their use does not extend to modeling the joint effect of a particular pattern of exposures.

### Estimation of SCMMs

The parameters of SCMMs can be estimated as the solution to GEEs (10). In estimation with GEEs, care should be taken to avoid biases that can arise, which we call “GEE bias.” In particular, the GEE estimates of the parameters in model (1) are unbiased only under the assumption that $Y_{t}$ is independent of future exposures and covariates conditional on past exposures and covariates for all t=1,…,T (11); $E\left(Y_{t}|{\bar{X}}_{t},{\bar{L}}_{t}\right)=E\left(Y_{t}|{\bar{X}}_{T},{\bar{L}}_{T}\right)$. See Web Appendix 1 (available at https://academic.oup.com/aje) for further discussion. Such biases can be avoided either by using an independence working correlation matrix or, preferably, by including prior outcomes in the regression model, the latter being more efficient:

(3) $$\begin{array}{c} E\left(Y_{t}|{\bar{X}}_{t},{\bar{L}}_{t},{\bar{Y}}_{t-1}\right)=\beta_{0}+\beta_{X1}X_{t}+\beta_{X2}X_{t-1}\\ +\beta_{L}^{T}L_{t}+\beta_{Y}Y_{t-1}. \end{array}$$

Including the outcome history in the model is not only desirable to increase precision but often also necessary when, as in Figure 1B, the outcome history confounds the association between $X_{t}$ and $Y_{t}$. We recommend adjustment for prior outcomes in the SCMM.

### Incorporating propensity scores

It may be advantageous to include adjustment for propensity scores in the SCMM. The propensity score for an individual at time t is their probability of having the exposure at time t conditional on the past:

(4) $$PS_{t}=\mathrm{Pr}\left(X_{t}=1|{\bar{X}}_{t-1},{\bar{L}}_{t},{\bar{Y}}_{t-1}\right).$$

One possible model for the propensity score is:

(5) $$PS_{t}=\frac{\exp(\rho_{0}+\rho_{X}X_{t-1}+\rho_{L}^{T}L_{t}+\rho_{Y}Y_{t-1})}{1+\exp(\rho_{0}+\rho_{X}X_{t-1}+\rho_{L}^{T}L_{t}+\rho_{Y}Y_{t-1})},$$

which can be fitted using logistic regression across all time points combined. The estimated propensity scores, PS^t, are then included in the SCMM:

(6) E(Yt|X¯t,L¯t,Y¯t−1)=β0+βX1Xt+βX2Xt−1+βLTLt+βYYt−1+βPSPS^t.

The propensity score model should include all variables suspected predictors of both $X_{t}$ and $Y_{t}$. Using propensity scores gives two primary advantages (12). First, in linear models it delivers a doubly robust estimate of the exposure effect $\beta_{X1}$, which is unbiased (in large samples) if either the SCMM (3) or the propensity score model (6) is correctly specified. Second, it down-weights exposed individuals for whom no comparable unexposed individuals can be found, and vice versa, thus avoiding model extrapolation when there is little overlap in the covariate distributions of exposed and unexposed individuals.

### IPW estimation of MSMs

This approach is also based on regression. MSMs are usually expressed in terms of an expected counterfactual outcome. We define $Y_{t}^{{\bar{x}}_{t}}$ to be the counterfactual outcome at time t for an individual, had there been an intervention by which their exposure history up to time t was ${\bar{X}}_{t}={\bar{x}}_{t}$. A MSM must correctly specify all treatment effects of interest, including long-term direct effects. Under the scenario in Figure 1, there are direct effects of $X_{t}$ and $X_{t-1}$ on $Y_{t}$, implying the MSM:

(7) $$E\left(Y_{t}^{{\bar{x}}_{t}}\right)=\omega_{0}+\omega_{X1}x_{t}+\omega_{X2}x_{t-1}.$$

Parameters of MSMs are estimated using IPW, in which the regression model implied by the MSM is fitted with the contribution of each individual weighted by the inverse probability of their observed exposures given their other covariates. Cole and Hernán (13) give overviews of the construction of weights. The estimation can be performed using weighted GEEs. GEE bias can be avoided by using an independence working correlation matrix. Unlike SCMMs, MSMs do not accommodate control for outcome history via regression adjustment; hence GEE bias cannot be avoided by adjustment for the outcome history (14, 15).

If interest is only in a short-term treatment effect, it is sufficient to specify a MSM based only on the short-term effect,

(8) $$E\left(Y_{t}^{x_{t}}\right)=\omega_{0}^{⁎}+\omega_{X1}^{⁎}x_{t},$$

provided that the confounding by past treatment $X_{t-1}$ is accounted for in the weights, by using unstabilized weights or by excluding past treatment from the numerator of the stabilized weights.

SCMMs can also be expressed in terms of counterfactuals; for example, model (3) can be written as

(9) $$\begin{array}{c} E\left(Y_{t}^{{\bar{x}}_{t}}|X_{t}=x_{t},{\bar{X}}_{t-1},{\bar{L}}_{t},{\bar{Y}}_{t-1}\right)\\ =\beta_{0}+\beta_{X1}x_{t}+\beta_{X2}X_{t-1}+\beta_{L}^{T}L_{t}+\beta_{Y}Y_{t-1}, \end{array}$$

and the propensity score could also be included.

### Comparison of estimands using SCMMs and IPW estimation of MSMs

MSM (7) and (8) parameterize the short-term effect of interest respectively as:

(10) $$\omega_{X1}=E(Y_{t}^{\left({\bar{x}}_{t-1},1\right)}-Y_{t}^{\left({\bar{x}}_{t-1},0\right)})$$

(11) $$\omega_{X1}^{⁎}=E(Y_{t}^{\left({\bar{X}}_{t-1},1\right)}-Y_{t}^{\left({\bar{X}}_{t-1},0\right)}).$$

Both are marginal effects. In contrast, in SCMM (3), the short-term effect is the conditional effect:

(12) $$\beta_{X1}=\;E\left(Y_{t}^{\left({\bar{x}}_{t-1},1\right)}-Y_{t}^{\left({\bar{x}}_{t-1},0\right)}|{\bar{X}}_{t-1}={\bar{x}}_{t-1},{\bar{L}}_{t},{\bar{Y}}_{t-1}\right).$$

For linear models $\beta_{X1}$, $\omega_{X1}$, and $\omega_{X1}^{⁎}$ all represent the same estimand, provided the MSMs and SCMM are correctly specified. For nonlinear models this no longer remains true due to noncollapsibility. In linear SCMMs, $\beta_{X1}$ in model (6) (including the propensity score) and in model (3) (excluding the propensity score) represents the same conditional effect provided either the propensity score model or the SCMM excluding the propensity score is correctly specified. Interestingly, this holds even if the functional form of the propensity score used in the SCMM is misspecified, provided the exposure effect is the same across all levels of the propensity score and the remaining predictors in the model (12).

MSMs can be used to estimate marginal effects or effects that are conditional on baseline variables. Stabilized weights can be used to fit only MSMs that condition on predictors used in the numerator of the weights; variables in the numerator should be incorporated as adjustment variables in the MSM. In our context, past exposure $X_{t-1}$ can be considered a baseline variable and included in the numerator of the stabilized weights, provided the MSM also includes that variable (as in MSM (7)). Unstabilized weights are most commonly used to estimate marginal effects, although they can also be used in fitting MSMs that condition on baseline variables.

### Extensions

#### Interactions

Because SCMMs estimate conditional effects, they extend straightforwardly to allow interactions between exposure and time-dependent covariates. If interactions exist, these should be incorporated into the SCMM. Failure to do so will result in a misspecified SCMM. In SCMMs including the propensity score, interactions between the covariate and the propensity score should be included for every covariate-exposure interaction. For example, to incorporate interactions between $X_{t}$ and $L_{t}$ and between $X_{t}$ and $Y_{t-1}$:

(13) E(Yt|X¯t,L¯t,Y¯t−1)=β0+βX1Xt+βX2Xt−1+βLTLt+βYYt−1+βPSPS^t+ηX1XtLt+ηX2XtYt−1+ηPS1PS^tLt+ηPS2PS^tYt−1.

Standard MSMs as described previously in this paper do not accommodate interactions between the exposure and time-dependent covariates because time-dependent confounders are handled in the weights rather than by adjustment. If interactions are present, MSMs are, however, still valid because they estimate marginal effects. “History-adjusted MSMs” (HA-MSMs) have been described that accommodate interactions with time-dependent covariates; these assume a MSM at each time point and model the counterfactual outcome indexed by treatment that occurs after that time point, conditional on some subset of the observed history up to that time (16, 17). However, HA-MSMs have not been much used in practice, and their validity remains in question (18).

Both MSMs and SCMMs can incorporate interactions between exposure and baseline variables.

#### Continuous exposures

SCMMs easily handle continuous exposures $X_{t}$ because they use standard regression. In linear SCMMs with a continuous exposure, it is advantageous to include adjustment for the propensity score, for the same reasons as discussed for a binary exposure, where here the propensity score is $PS_{t}\;=\;E\left(X_{t}|{\bar{X}}_{t-1},{\bar{L}}_{t},{\bar{Y}}_{t-1}\right)$ (12). In theory, IPW estimation of MSMs extends to continuous exposures by specifying a model for the conditional distribution of the continuous exposure in the weights. Different ways of constructing these weights have been compared (5), however the method has been found not to work well (4). A major concern is that correct specification of the entire distribution is difficult, and slight misspecification of the tails could have a big impact on the weights.

#### Binary and survival outcomes

For a binary outcome $Y_{t}$, the SCMM (e.g., model (3)) can be replaced by a logistic model. Propensity score adjustment is also advantageous in logistic SCMMs (12), ensuring double robustness for the test of no exposure effect. Logistic MSMs can also be used.

SCMMs excluding the propensity score deliver a conditional odds ratio while MSMs deliver unconditional odds ratios; for a binary outcome, these are different effects. SCMMs including the propensity score estimate a different conditional effect. All of these effects may be viewed as “causal.” A conditional effect is sometimes of most realistic interest, in particular when the exposed and unexposed are very different in their covariate histories. In that case, the observed data may carry insufficient information to infer the average outcome if everyone versus no one were exposed, while there may be sufficient information to answer that question for subgroups where there is sufficient overlap (12, 19).

SCMMs and IPW estimation of MSMs can also be used to study short-term exposure effects in a survival analysis setting using Cox regression, using exposures and covariates measured at scheduled visits (20). This is an area for further work.

## A TEST FOR LONG-TERM DIRECT EFFECTS

SCMMs give insight into total exposure effects. However, it is useful to understand whether earlier exposures directly affect a subsequent outcome other than via intermediate exposures. Focusing on Figure 1B, we outline a test for the existence of any direct effect of $X_{t-1}$ on $Y_{t}$, except that mediated through $X_{t}$. This long-term direct effect is represented by unblocked pathways from $X_{t-1}$ to $Y_{t}$ that do not pass through $X_{t}$.

The test uses the following steps:

**Step 1.** Fit a SCMM for $Y_{t}$ given $X_{t}$ and the covariate history up to time t, including prior exposures and outcomes. This is used to infer the short-term effect of $X_{t}$ on $Y_{t}$.
**Step 2.** Using the model from step 1, obtain the predicted outcomes ${\hat{Y}}_{t}$ when $X_{t}=0\;(t=1,\ldots ,T)$ (i.e., when we force no effect of $X_{t}$ on $Y_{t}$).
**Step 3.** The test of interest is now a test of the hypothesis that ${\hat{Y}}_{t}$ is independent of $X_{t-1}$ given the covariate history up to time t−1. This hypothesis can be tested by fitting a model for $X_{t-1}$ given the covariate history up to time t−1 and ${\hat{Y}}_{t}$; for example, for a binary exposure we would test the hypothesis that $\delta_{Y}=0$ in the model:

(14) $$\begin{array}{c} E\left(X_{t-1}|{\bar{X}}_{t-2},{\bar{L}}_{t-1},{\hat{Y}}_{t},{\bar{Y}}_{t-2}\right)\;\\ =\;\frac{\exp(\delta_{0}+\delta_{X}^{T}{\bar{X}}_{t-2}+\delta_{L}^{T}{\bar{L}}_{t-1}+\delta_{\bar{Y}}^{T}{\bar{Y}}_{t-2}+\delta_{Y}{\hat{Y}}_{t})}{1+\exp(\delta_{0}+\delta_{X}^{T}{\bar{X}}_{t-2}+\delta_{L}^{T}{\bar{L}}_{t-1}+\delta_{\bar{Y}}^{T}{\bar{Y}}_{t-2}+\delta_{Y}{\hat{Y}}_{t})}. \end{array}$$

This is fitted across all visits combined.

The usual estimate of the standard error of ${\hat{\delta }}_{Y}$ will be erroneously small because it ignores that the ${\hat{Y}}_{t}$ are predicted values. We therefore propose using bootstrapping.

Robins (21) proposed the direct effect g-null test, which is readily applicable to test for the presence of long-term direct effects. Relative to the Robins test, our proposed test has the advantage of not relying on inverse probability weighting and thus being more naturally suited to handling continuous exposures. Our test, as described so far, assesses the presence of long-term direct effects when setting $x_{t}$ to 0; it will generally be a good idea to additionally assess whether there is evidence for long-term direct effects when setting $x_{t}$ to values other than zero.

## SIMULATION STUDY

We used simulation studies to compare SCMMs with IPW estimation of MSMs for the short-term effect of a binary exposure $X_{t}$ on a continuous outcome $Y_{t}$, and to assess the performance of the test for long-term direct effects. Data were simulated according to Figure 1A, using n=200 individuals observed at T=5 visits (simulation scenario 1). To further assess the test for long-term direct effects we generated data under a second scenario in which there is no direct effect of $X_{t-1}$ on $Y_{t}$ ($\delta_{Y}=0$ in model (14)), represented by a modification of Figure 1A with the arrows from $X_{t-1}$ to $Y_{t}$ removed (simulation scenario 2). See Web Appendix 2 for details.

### Methods

In each simulated data set under scenario 1, we fitted SCMMs and MSMs using GEEs with independent and unstructured working correlation matrices. We considered different forms for the SCMMs and MSMs to illustrate earlier points on model misspecification and GEE bias.

The effect of $X_{t}$ on $Y_{t}$ is confounded by prior exposure $X_{t-1}$ and prior outcome $Y_{t-1}$ (via *U*_*Y*_), implying that to obtain an unbiased effect estimate, the SCMM should either include $X_{t-1}$ and $Y_{t-1}$, or it should include $X_{t-1}$ and use an unstructured working correlation matrix. To illustrate the main points we considered four SCMMs: i) $E\left(Y_{t}|{\bar{X}}_{t},{\bar{Y}}_{t-1}\right)=\beta_{0}+\beta_{X1}X_{t}$; ii) $E\left(Y_{t}|{\bar{X}}_{t},{\bar{Y}}_{t-1}\right)=\beta_{0}+\beta_{X1}X_{t}+\beta_{Y}Y_{t-1}$; iii) $E\left(Y_{t}|{\bar{X}}_{t},{\bar{Y}}_{t-1}\right)=\beta_{0}+\beta_{X1}X_{t}+\beta_{X2}X_{t-1}$; and iv) $E\left(Y_{t}|{\bar{X}}_{t},{\bar{Y}}_{t-1}\right)=\beta_{0}+\beta_{X1}X_{t}+\beta_{X2}X_{t-1}+\beta_{Y}Y_{t-1}$. The same SCMMs were fitted with adjustment for the propensity score. The propensity score model for $X_{t}$ included $Y_{t-1}$ and $X_{t-1}$.

To estimate a total effect using IPW estimation of MSMs, the MSM should either correctly model the effect of exposures on the outcome up to and including the exposure whose total effect we wish to estimate (model (7)), or it should correctly model the effect of the exposure whose total effect we wish to estimate (model (8)) and incorporate confounding by past exposures in the weights. The models used to construct the weights should include all confounders of the association between $X_{t}$ and $Y_{t}$, including prior exposures and outcomes. We considered two MSMs: 1) $E\left(Y_{t}^{x_{t}}\right)=\omega_{0}^{⁎}+\omega_{X1}^{⁎}x_{t}$; and 2) $E\left(Y_{t}^{{\bar{x}}_{t}}\right)=\omega_{0}+\omega_{X1}x_{t}+\omega_{X2}x_{t-1}$. Unstabilized and stabilized weights were used and obtained using logistic regression models fitted across all 5 visits. In the weight denominators, we used a logistic model for $X_{t}$ with $X_{t-1}$ and $Y_{t-1}$ as predictors. In the numerator of the stabilized weights, we used a logistic model for $X_{t}$ with $X_{t-1}$ as the predictor. Unstabilized weights are not recommended because they are known to be highly variable, but we include them for comparison. It has been suggested that weights could be truncated to improve precision (13). We consider stabilized weights with truncation of the p% smallest and largest weights (p=1,5,10,20).

The test for long-term direct effects was performed in simulation scenarios 1 and 2. In Step 1 we fitted a SCMM of the form $E\left(Y_{t}|{\bar{X}}_{t},{\bar{Y}}_{t-1}\right)\;=\;\beta_{0}\;+\;\overset{4}{\underset{j=0}{\sum }}\beta_{Xj}X_{t-j}+\overset{4}{\underset{j=0}{\sum }}\beta_{Yj}Y_{t-j}$, where $X_{t}$ and $Y_{t}$ are set to zero for t≤0. The model fitted in Step 3 was as in model (14) using all lags of X and Y (omitting ${\bar{L}}_{t-1}$). A 95% confidence interval for $\delta_{Y}$ was estimated using 1,000 bootstrap samples, using the percentile method (22, 23). We obtained the percentage of the 1,000 bootstrap 95% confidence intervals (23) that excluded 0. A *P* value for a 2-sided test of the null hypothesis could be obtained as the number of bootstrapped estimates of $\delta_{Y}$ that lie more than a distance $\left|{\hat{\delta }}_{Y}\right|$ from 0, divided by the number of bootstrap samples, which should be large to capture small *P* values.

### Simulation results

#### Comparison of results from SCMMs and IPW estimation of MSMs

Results are shown in Table 1. In the SCMMs, model i fails to account for confounding by $X_{t-1}$ and $Y_{t-1}$, and model ii fails to account for confounding by $X_{t-1}$; in neither case can this by accounted for using an unstructured working correlation matrix, which only handles confounding by $Y_{t-1}$. Hence SCMMs i and ii give biased effect estimates. Model iii, fitted using an independence working correlation matrix, fails to account for confounding by $Y_{t-1}$, resulting in bias. However, the bias is eliminated by using an unstructured working correlation matrix. The analysis under model iii based on a nonindependence working correlation structure would nonetheless be subject to confounding bias and GEE bias when that working correlation structure is misspecified, as is likely when the outcome model is nonlinear. Model iv accounts for both sources of confounding directly, giving unbiased effect estimates using any form for the working correlation matrix. We recommend SCMM iv with an independence working correlation structure. Propensity score adjustment delivers a double-robustness property and therefore gives unbiased estimates under all models using any working correlation matrix.

**Table 1.** Results of Simulation Studies to Compare Sequential Conditional Mean Models with Inverse Probability Weighted Estimation of Marginal Structural Models

Model^a^	Independence			Unstructured		
	Bias^b^	95% CI^c^	SD^d^	Bias^b^	95% CI^c^	SD^d^
SCMM						
Form of $E\left(Y_{t}|{\bar{X}}_{t},{\bar{Y}}_{t-1}\right)$						
i) $\beta_{0}+\beta_{X1}X_{t}$	0.425	0.420, 0.430	0.081	0.256	0.251, 0.262	0.087
ii) $\beta_{0}+\beta_{X1}X_{t}+\beta_{Y}Y_{t-1}$	0.151	0.146, 0.156	0.080	0.050	0.045, 0.055	0.086
iii) $\beta_{0}+\beta_{X1}X_{t}+\beta_{X2}X_{t-1}$	0.115	0.109, 0.120	0.092	−0.002	0.008, 0.004	0.095
iv) $\beta_{0}+\beta_{X1}X_{t}+\beta_{X2}X_{t-1}+\beta_{Y}Y_{t-1}$	−0.001	−0.007, 0.005	0.095	0.001	−0.004, 0.007	0.095
SCMM using propensity scores						
Form of $E\left(Y_{t}|{\bar{X}}_{t},{\bar{Y}}_{t-1}\right)$						
i) β0+βX1Xt+βPSPS^t	0.001	−0.005, 0.007	0.096	0.001	−0.005, 0.007	0.095
ii) β0+βX1Xt+βYYt−1+βPSPS^t	0.001	−0.005, 0.007	0.096	0.006	0.000, 0.012	0.097
iii) β0+βX1Xt+βX2Xt−1+βPSPS^t	0.003	−0.002, 0.009	0.096	−0.002	−0.008, 0.004	0.095
iv) β0+βX1Xt+βX2Xt−1+βYYt−1+βPSPS^t	−0.001	−0.007, 0.005	0.096	0.001	−0.005, 0.007	0.096
IPW estimation of MSMs						
Unstabilized weights						
i) $E\left(Y_{t}^{x_{t}}\right)=\omega_{0}^{⁎}+\omega_{X1}^{⁎}x_{t}$	0.022	0.001, 0.043	0.340	0.046	−0.137, 0.230	2.959
ii) $E\left(Y_{t}^{{\bar{x}}_{t}}\right)=\omega_{0}+\omega_{X1}x_{t}+\omega_{X2}x_{t-1}$	0.007	−0.012, 0.026	0.306	3.635	−3.208, 10.478	110.4
Stabilized weights						
i) $E\left(Y_{t}^{x_{t}}\right)=\omega_{0}^{⁎}+\omega_{X1}^{⁎}x_{t}$	0.297	0.291, 0.302	0.090	0.187	0.180, 0.194	0.110
ii) $E\left(Y_{t}^{{\bar{x}}_{t}}\right)=\omega_{0}+\omega_{X1}x_{t}+\omega_{X2}x_{t-1}$	−0.002	−0.009, 0.004	0.107	−0.060	−0.067, −0.053	0.114
Stabilized weights: truncated at the 1st and 99th percentiles						
i) $E\left(Y_{t}^{x_{t}}\right)=\omega_{0}^{⁎}+\omega_{X1}^{⁎}x_{t}$	0.309	0.304, 0.315	0.087	0.196	0.190, 0.202	0.098
ii) $E\left(Y_{t}^{{\bar{x}}_{t}}\right)=\omega_{0}+\omega_{X1}x_{t}+\omega_{X2}x_{t-1}$	0.018	0.012, 0.024	0.101	−0.051	−0.058, −0.045	0.106
Stabilized weights: truncated at the 5th and 95th percentiles						
i) $E\left(Y_{t}^{x_{t}}\right)=\omega_{0}^{⁎}+\omega_{X1}^{⁎}x_{t}$	0.325	0.320, 0.330	0.086	0.214	0.209, 0.220	0.092
ii) $E\left(Y_{t}^{{\bar{x}}_{t}}\right)=\omega_{0}+\omega_{X1}x_{t}+\omega_{X2}x_{t-1}$	0.025	0.019, 0.032	0.099	−0.043	−0.049, −0.037	0.102
Stabilized weights: truncated at the 10th and 90th percentiles						
i) $E\left(Y_{t}^{x_{t}}\right)=\omega_{0}^{⁎}+\omega_{X1}^{⁎}x_{t}$	0.341	0.335, 0.346	0.085	0.225	0.219, 0.230	0.091
ii) $E\left(Y_{t}^{{\bar{x}}_{t}}\right)=\omega_{0}+\omega_{X1}x_{t}+\omega_{X2}x_{t-1}$	0.044	0.038, 0.050	0.097	−0.032	−0.039, −0.026	0.100
Stabilized weights: truncated at the 20th and 80th percentiles						
i) $E\left(Y_{t}^{x_{t}}\right)=\omega_{0}^{⁎}+\omega_{X1}^{⁎}x_{t}$	0.364	0.359, 0.370	0.083	0.236	0.231, 0.242	0.088
ii) $E\left(Y_{t}^{{\bar{x}}_{t}}\right)=\omega_{0}+\omega_{X1}x_{t}+\omega_{X2}x_{t-1}$	0.067	0.061, 0.073	0.094	−0.021	−0.027, −0.015	0.097

Abbreviations: CI, confidence interval; GEE, generalized estimating equation; IPW, inverse probability weight; MSM, marginal structural model; SCMM, sequential conditional mean model; SD, standard deviation.

^a^ All models were fitted using GEEs with an independence working correlation matrix and an unstructured working correlation matrix.

^b^ Bias in the estimated short-term causal effect of $X_{t}$ on $Y_{t}$ averaged over 1,000 simulations.

^c^ Monte Carlo 95% confidence interval corresponding to the bias.

^d^ Empirical standard deviation of the estimates.

MSM 1 ignores the direct effect of $X_{t-1}$ on$\;Y_{t}$; this can be accounted for using unstabilized weights but not stabilized weights. There is some small finite sample bias using unstabilized weights. In practice, bias can also occur due to lack of positivity, which requires both exposed and unexposed individuals at every level of the confounders (13). MSM 2 is correctly specified, and the estimates are unbiased using either stabilized weights or unstabilized weights. As expected, unstabilized weights (Web Appendix 3 and Web Table 1) give large empirical standard deviations, especially using an unstructured working correlation matrix. Stabilized weights improve precision, but the empirical standard deviations remain larger than under SCMMs. Precision was improved under truncation but comes at a cost of bias, which is small using MSM 2 but quite large using MSM 1. Using an unstructured working correlation matrix gives GEE bias; this is true for both unstabilized and stabilized weights, but it is not evident here for unstabilized weights due to large empirical standard deviations.

Web Table 2 shows results for 10 study visits, when the efficiency of IPW estimation of MSMs compared with SCMMs is further reduced. Results from additional simulation scenarios (see Web Figure 1) are given in Web Appendix 4 and Web Table 3. Simulations did not include time-varying covariates $L_{t}$: Differences in precision of estimates from the two approaches will generally be greater in this case.

#### Results from the test for long-term direct effects

In scenario 1, the mean estimate of $\delta_{Y}$ across 1,000 simulations was 7.253 (standard deviation, 1.854), and 99.7% of the 95% confidence intervals for $\delta_{Y}$ excluded 0, indicating evidence against the null hypothesis of no long-term direct effect. In scenario 2, the mean estimate of $\delta_{Y}$ was 0.012 (standard deviation, 1.102), and 5.2% of the 95% confidence intervals for $\delta_{Y}$ excluded 0, demonstrating approximately correct type I errors.

## DISCUSSION

We have shown how standard regression methods using SCMMs can be used to estimate total effects of a time-varying exposure on a subsequent outcome by controlling for confounding by prior exposures, outcomes, and time-varying covariates. We compared this with IPW estimation of MSMs, which handles time-varying confounding when estimating joint effects but which can also be used to estimate total effects. Other methods for estimating joint effects include g-estimation and g-computation (see Daniel et al. (3) for an overview), which have not been used extensively in practice (24–26). There is a close connection between SCMMs and structural nested mean models (SNMMs) (26), in which a parametric model is specified for the causal effect of interest among people receiving a given level of treatment (e.g., $g\left\{E\left(Y^{\left({\bar{x}}_{t-1},1\right)}|{\bar{X}}_{t}={\bar{x}}_{t},{\bar{L}}_{t}\right)\right\}-g\left\{E\left(Y^{\left({\bar{x}}_{t-1},0\right)}|{\bar{X}}_{t}={\bar{x}}_{t},{\bar{L}}_{t}\right)\right\}$). In linear models, our propensity score adjusted estimates are equivalent to efficient g-estimates in a SNMM for short-term effects (27). When the remaining long-term direct effects are of interest, estimation in linear SNMMs becomes more involved, but it is still feasible using standard software (27, 28).

There is a large literature on adjustment for baseline outcomes in studies of the relationship between an exposure and a follow-up outcome or change in outcome. Glymour et al. (29) presented challenges arising in this setting in a causal context. Key differences between that setting and ours are that we focused on repeated measures of exposures, covariates, and outcomes, and we used adjustment for all relevant past measures in order to estimate a total effect.

A total effect may be the most realistic effect of interest. It could be particularly informative to estimate the total effect of an exposure at a given time on outcomes at a series of future times. We outlined a new test for existence of long-term direct effects, which may be used as a simple alternative to the direct effect g-null test. If the test provides no evidence for existence of long-term direct effects, this informs the investigator that joint exposure effects can be estimated without the need for complex methods.

SCMMs estimate conditional effects, whereas MSMs are typically used to estimate marginal effects. In linear models without interactions, the conditional and unconditional effects coincide but are otherwise different. Conditional effects may be more realistic for interpretation, in particular when the exposed and unexposed have quite different covariate histories.

Misspecification of SCMMs can lead to confounding bias. Without strong prior information, we must assume many possible associations, including long-term direct effects, and include adjustment for prior exposures, outcomes, and covariates. We recommend adjustment for the outcome history and propensity scores, and estimation using independence GEE. SCMMs adjusting for the propensity score are less vulnerable to misspecification than MSMs because of their double-robustness property. However, unlike MSMs, SCMMs require correct modeling of interactions of the exposure with the covariate history. SCMMs give better precision even than stabilized weights in realistic scenarios. In addition to their simplicity and familiarity, SCMMs extend more easily to accommodate continuous exposures, drop-out, and missing data (see Web Appendix 5).

## Supplementary Material

Web Material

## Abbreviations

GEE: generalized estimating equation
IPW: inverse probability weight
MSM: marginal structural model
SCMM: sequential conditional mean model
