# Supplementary material for: Analysis of Longitudinal Studies With Repeated Outcome Measures: Adjusting for Time-Dependent Confounding Using Conventional Methods
Source: Am J Epidemiol. 2017 Sep 6;187(5):1085–92. doi: 10.1093/aje/kwx311 (PMC5928464; doi:10.1093/aje/kwx311)
Supplement: Web Material [file kwx311keoghwebmaterialfinal.pdf]

Web Materials

Analysis of longitudinal studies with repeated outcome measures: Adjusting for time-dependent confounding using conventional methods

Web Appendix 1 Estimation in SCMMs using GEEs

As noted in the main text, the GEE estimates of the parameters in SCMM (1) are only unbiased under the assumption that Y_t is independent of future exposures and covariates conditional on past exposures and covariates for all $t = 1, \ldots, T$ [1]:

$$E(Y_t|\bar{X}_t, \bar{\mathbf{L}}_t) = E(Y_t|\bar{X}_T, \bar{\mathbf{L}}_T). \tag{A1}$$

This assumption is only met in certain circumstances. Referring to Figure 1(b), the assumption is *not* met when:

(a) There is a direct arrow from Y_{t-1} to X_t (as shown).

(b) There is a direct arrow from Y_{t-1} to $\mathbf{L}_t$ (as shown).

(c) The association between Y_{t-1} and $\mathbf{L}_t$ is confounded by U_Y (not shown in the figure).

(d) There is unmeasured confounding between Y_{t-1} and X_t (not shown in the figure).

We refer to bias induced by a violation of (A1) as *GEE bias*. However, we emphasise that this is a specific type of bias which can arise when the assumption in (A1) is violated, and is not a bias from which all GEEs suffer. One way to avoid GEE bias is to solve the GEEs using a diagonal working correlation matrix which assumes independence between the outcome measures made at different times [1] ([2] and [3] discuss more efficient alternatives). The resulting estimates can be inefficient because they ignore information on which observations were obtained from which subject. This can be remedied by explicitly modelling the dependence between the repeated outcomes as described in the main text (equation (2)).

Web Appendix 2 Simulation study: Data generation

Simulation Scenario 1

We generated data for $n = 200$ individuals observed at $T = 5$ visits based on the scenario illustrated in Figure 1(a). The data were simulated by first generating X_1, followed by generating Y_t to depend on X_t, X_{t-1} and U_Y ($t = 1, \ldots, T$), and finally generating X_t to depend on X_{t-1}, Y_{t-1} and U_X ($t = 2, \ldots, T$). The exposure at visit 1 for individual i, X_{1i}, was generated from a Bernoulli distribution using the model $\text{Pr}(X_{1i} = 1) = e^{\alpha_{01}+u_{Xi}}/(1+e^{\alpha_{01}+u_{Xi}})$ where $e^{\alpha_{01}} = 1/3$ and where the u_{Xi} are individual random effects generated from a normal distribution with mean 0 and standard deviation 0.2. An individual with the mean random effect ($u_{Xi} = 0$) has probability 1/4 of having $X_{1i} = 1$ at visit 1.

The continuous outcome for individual i at visit t, Y_{ti} ($t = 1, \ldots, 5$), was generated using

$$Y_{ti} = X_{ti} + 0.5X_{t-1,i} + u_{Yi} + \epsilon_{Y_{ti}}, t = 1, \ldots, 5 \tag{A2}$$

where the u_{Yi} are random effects generated from a normal distribution with mean 0 and standard deviation 0.5 and where $\epsilon_{Y_{ti}}$ are random errors generated from a standard normal distribution. The parameter that represents the effect of X_t on Y_t has true value 1 and it is this effect that we aim to estimate.

Exposures at subsequent visits ($t = 2, \ldots, T$) were generated using

$$\Pr(X_{ti} = 1|\bar{X}_{t-1,i}, \bar{Y}_{t-1,i}) = \frac{\exp(\alpha_{0t} + \alpha_X X_{t-1,i} + \alpha_Y Y_{t-1,i} + u_{Xi})}{1 + \exp(\alpha_{0t} + \alpha_X X_{t-1,i} + \alpha_Y Y_{t-1,i} + u_{Xi})} \quad \text{(A3)}$$

using $e^{\alpha_Y} = 2$, $\alpha_{0t} = \log(0.2/0.8)$, $\alpha_X = \log(0.2/0.8) - \alpha_{0t}$. The probability of being exposed at time t ($t \geq 2$) is 0.2 for an individual who was unexposed at time $t - 1$ and 0.8 for an individual exposed at time $t - 1$, for a person with average random effect and $Y_{t-1} = 0$.

Simulation Scenario 2

A second simulation scenario was used to further assess the test for long term direct exposure effects. Scenario 2 is a modification of Scenario 1 with the direct effect of X_{t-1} on Y_t omitted, by omitting the term in X_{t-1} from equation (A2).

1000 data sets were simulated.

Web Appendix 3 Further results from simulation scenario 1

Table 1 summarises the weights used in the IPW estimation of MSM in simulation scenario 1. Table 2 shows results from extending simulation scenario 1 to 10 visits per individual. When the number of visits is increased the observed biases are seen to be greater.

Web Table 1: Simulation Scenario 1. Summary of Weights From IPW Analyses Corresponding to the Results Shown in Table 1. The Results Shown are the Mean Across 1000 Simulated Data Sets of the Mean of the Weights in a Given Simulation, the SD of the Weights, the Median of the Weights, and the Minimum and Maximum Weights.

Weights	Mean weight	SD weight	Median weight	Min. weight	Max. weight
Unstabilized	12.348	63.428	3.610	1.339	1453.428
Stabilized	1.000	0.469	0.989	0.154	6.070

Web Table 2: Simulation Scenario 1. Simulation Results Extended to 10 Visits Per Person. The Results Shown are the Bias in the Estimated Short Term Causal Effect of X_t on Y_t Averaged over 1000 Simulations, the Corresponding Monte Carlo 95% Confidence Interval (in Brackets), and the Empirical Standard Deviation (SD). All Models Were Fitted Using GEEs with an Independence Working Correlation Matrix and an Unstructured Working Correlation Matrix.

Model	Independence		Unstructured	
	Bias (95% CI)	SD	Bias (95% CI)	SD
**Sequential conditional mean models**				
Form of $E(Y_t \vert \bar{X}_t, \bar{Y}_{t-1})$				
(i) X_t	0.514 (0.510,0.519)	0.068	0.294 (0.290,0.298)	0.064
(ii) $X_t + Y_{t-1}$	0.192 (0.188,0.196)	0.063	0.061 (0.057,0.065)	0.063
(iii) $X_t + X_{t-1}$	0.133 (0.129,0.138)	0.072	0.000 (-0.005,0.004)	0.070
(iv) $X_t + X_{t-1} + Y_{t-1}$	0.001 (-0.004,0.005)	0.072	0.006 (0.002,0.010)	0.071
**Sequential conditional mean models using propensity scores**				
Form of $E(Y_t \vert \bar{X}_t, \bar{Y}_{t-1}, \widehat{PS}_t)$				
(i) $X_t + \widehat{PS}_t$	0.009 (0.005,0.014)	0.073	0.004 (0.000,0.009)	0.071
(ii) $X_t + Y_{t-1} + \widehat{PS}_t$	0.006 (0.002,0.011)	0.073	0.014 (0.010,0.019)	0.072
(iii) $X_t + X_{t-1} + \widehat{PS}_t$	0.013 (0.008,0.018)	0.074	0.000 (-0.005,0.004)	0.070
(iv) $X_t + X_{t-1} + Y_{t-1} + \widehat{PS}_t$	0.002 (-0.003,0.006)	0.073	0.006 (0.002,0.010)	0.072
**IPW and MSMs**				
Form of $E(Y_t^{\bar{x}_t})$				
Unstabilized weights				
(i) $\omega_0 + \omega_{X1} x_t$	0.073 (0.042,0.104)	0.498	0.147 (-0.116,0.409)	4.236
(ii) $\omega_0 + \omega_{X1} x_t + \omega_{X2} x_{t-1}$	0.034 (0.005,0.062)	0.459	0.051 (-0.190,0.292)	3.893
Stabilized weights				
(i) $\omega_0 + \omega_{X1} x_t$	0.339 (0.334,0.343)	0.079	0.247 (0.231,0.264)	0.263
(ii) $\omega_0 + \omega_{X1} x_t + \omega_{X2} x_{t-1}$	-0.004 (-0.010,0.002)	0.092	-0.016 (-0.080,0.048)	1.025
Stabilized weights: truncated at the 1st and 99th percentiles				
(i) $\omega_0 + \omega_{X1} x_t$	0.354 (0.349,0.358)	0.074	0.235 (0.229,0.241)	0.096
(ii) $\omega_0 + \omega_{X1} x_t + \omega_{X2} x_{t-1}$	0.014 (0.009,0.020)	0.083	-0.055 (-0.063,-0.048)	0.123
Stabilized weights: truncated at the 5th and 95th percentiles				
(i) $\omega_0 + \omega_{X1} x_t$	0.374 (0.370,0.379)	0.072	0.253 (0.249,0.258)	0.073
(ii) $\omega_0 + \omega_{X1} x_t + \omega_{X2} x_{t-1}$	0.025 (0.020,0.030)	0.079	-0.043 (-0.048,-0.038)	0.081
Stabilized weights: truncated at the 10th and 90th percentiles				
(i) $\omega_0 + \omega_{X1} x_t$	0.393 (0.389,0.398)	0.070	0.263 (0.259,0.267)	0.070
(ii) $\omega_0 + \omega_{X1} x_t + \omega_{X2} x_{t-1}$	0.043 (0.038,0.048)	0.076	-0.034 (-0.039,-0.029)	0.077
Stabilized weights: truncated at the 20th and 80th percentiles				
(i) $\omega_0 + \omega_{X1} x_t$	0.424 (0.420,0.429)	0.068	0.275 (0.271,0.279)	0.067
(ii) $\omega_0 + \omega_{X1} x_t + \omega_{X2} x_{t-1}$	0.069 (0.065,0.074)	0.074	-0.022 (-0.027,-0.017)	0.074

Web Appendix 4 Results from simulation scenarios 2-4

The comparison of SCMMs and IPW of MSMs was also investigated in simulation scenario 2 (described above), and in two further scenarios:

Simulation scenario 3 No direct effect of Y_{t-1} on X_t.

Simulation scenario 4 No direct effect of X_{t-1} on Y_t and no direct effect of Y_{t-1} on X_t.

Simulation scenarios 2-4 are illustrated in Figure A1. SCMMs and IPW of MSMs were applied exactly as described for the simulation scenario 1 and the results are shown in Table A3. The test for long term direct effects was also performed.

Simulation scenario 2

Estimates from SCMMs (i) and (iii) are subject to confounding bias (Y_{t-1} acts as a confounder via U_Y) when an independence working correlation matrix is used. This bias is eliminated by using an unstructured working correlation matrix. Adjustment for Y_{t-1} under SCMM (ii) gives a biased estimate when using an independence working correlation matrix because adjusting for Y_{t-1} opens up a 'back-door' path from X_t to Y_t via U_Y, inducing confounding by X_{t-1} ('collider-stratification'). This bias is eliminated by using an unstructured working correlation matrix because the effect of modelling the correlation across outcomes is that the GEE estimates assign a zero coefficient to Y_{t-1}, thus effectively overcoming the earlier problem of collider-stratification. Model (iv) gives an unbiased estimate by inclusion of both X_{t-1} and Y_{t-1}.

Propensity score adjustment delivers a double robustness property and therefore gives unbiased estimates under all models in all scenarios, using either working correlation matrix.

MSMs (i) and (ii) are both correctly specified and both give almost unbiased estimates using either stabilized or unstabilized weights. As we expect, unstabilized weights give large empirical standard deviations, especially using an unstructured working correlation matrix. The empirical standard deviations are larger using stabilized IPW estimates than using SCMM. In this scenario using truncated weights results in some very small gains in efficiency, but at the expense of bias, and the IPW estimates still have lower efficiency than the SCMM estimates except under extreme truncation of the weights.

Simulation scenario 3

Here the effect of X_t on Y_t is confounded by X_{t-1}, therefore SCMMs (i) and (ii) give confounding bias. MSM (i) does not model the direct effect of X_{t-1} on Y_t; this can be accounted for using unstabilized weights and approximately unbiased estimates are obtained using unstabilized weights (there is some small finite sample bias). The direct effect of X_{t-1} on Y_t is not accounted for in MSM (i) fitted using stabilized weights, resulting in bias. MSM (ii) is correctly specified and the estimates are unbiased (apart from small finite sample bias). stabilized weights give similar precision as found using SCMMs. This is not surprising because the probabilities in the numerator and denominator of the stabilized weights are theoretically identical in this scenario, so all stabilized weights are close to 1. For this reason, truncating the stabilized weights has negligible impact on the results.

In the test for long term direct effects the mean estimate of δ_Y across 1000 simulations was 6.900 with standard deviation 1.666, and 99.7% of the 95% confidence intervals for δ_Y excluded 0.

Simulation scenario 4

Here there is no confounding of the effect of X_t on Y_t by past exposures or past outcome. Moreover, past outcome does not have a direct effect on future exposure, hence no GEE bias. SCMMs (i) and (iii) thus give unbiased estimates using both an independence and an unstructured working correlation matrix. As in Scenario 2, adjustment for Y_{t-1} under Model (ii) gives a biased estimate when using an independence working correlation matrix due to collider-stratification, with the bias being eliminated by using an unstructured working correlation matrix. Model (iv) gives an unbiased estimate by inclusion of X_{t-1}.

MSMs (i) and (ii) are both correctly specified and both give almost unbiased estimates using either stabilized or unstabilized weights (there is small finite sample bias for MSM (ii)). As we expect, unstabilized weights give large empirical standard deviations, especially using an unstructured working correlation matrix. Stabilized weights give similar precision as found using SCMMs. As in Scenario 3, this is not surprising because all stabilized weights are close to 1 and for this reason, truncating the stabilized weights has negligible impact on the results.

In the test for long term direct effects the mean estimate of δ_Y across 1000 simulations was 0.088 with standard deviation 2.357, and 7.1% of the 95% confidence intervals for δ_Y excluded 0.

Web Figure 1: Associations Between an Exposure (X_t) and Outcome (Y_t) Measured Longitudinally, With Random Effects U_X and U_Y.

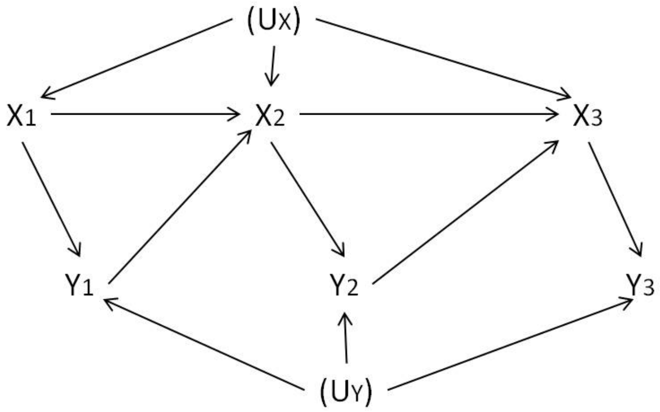

(a) Simulation scenario 2

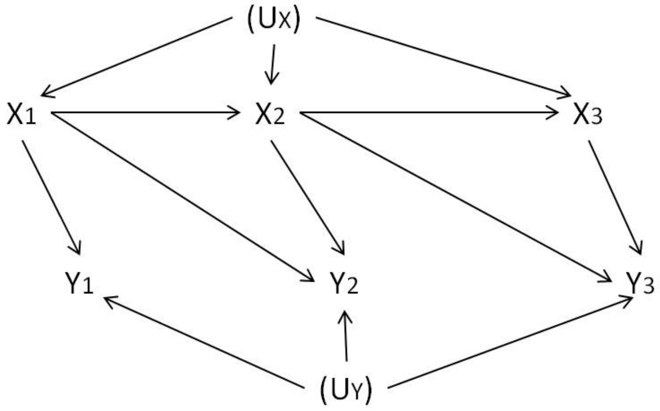

(b) Simulation scenario 3

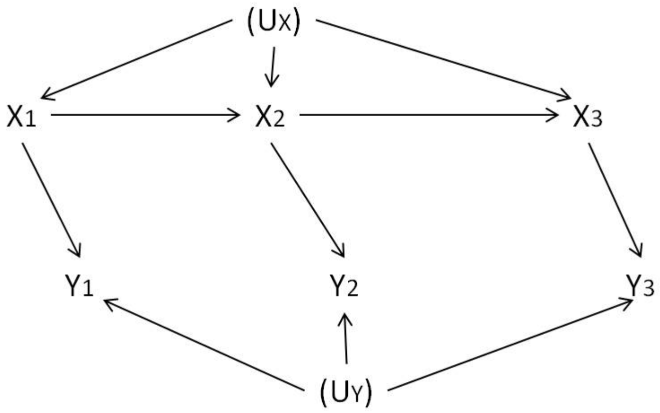

(c) Simulation scenario 4

Web Table 3: Results from Simulation Scenarios 2-4. Simulation Results. The Results Shown are the Bias in the Estimated Short Term Causal Effect of X_t on Y_t Averaged over 1000 Simulations, the Corresponding Monte Carlo 95% Confidence Interval (in Brackets), and the Empirical Standard Deviation (SD). All Models Were Fitted Using GEEs with an Independence Working Correlation Matrix and an Unstructured Working Correlation Matrix.

Model	Independence		Unstructured	
	Bias (95% CI)	SD	Bias (95% CI)	SD
Simulation scenario 2				
**Sequential conditional mean models**				
Form of $E(Y_t\|\bar{X}_t, \bar{Y}_{t-1})$				
(i) X_t	0.132 (0.127,0.137)	0.078	0.000 (-0.005,0.005)	0.079
(ii) $X_t + Y_{t-1}$	-0.054 (-0.060,-0.049)	0.081	-0.001 (-0.007,0.005)	0.094
(iii) $X_t + X_{t-1}$	0.117 (0.111,0.123)	0.090	-0.001 (-0.007,0.004)	0.093
(iv) $X_t + X_{t-1} + Y_{t-1}$	0.000 (-0.006,0.006)	0.093	-0.001 (-0.007,0.005)	0.093
**Sequential conditional mean models using propensity scores**				
Form of $E(Y_t\|\bar{X}_t, \bar{Y}_{t-1}, \widehat{PS}_t)$				
(i) $X_t + \widehat{PS}_t$	0.000 (-0.005,0.006)	0.094	-0.001 (-0.007,0.005)	0.094
(ii) $X_t + Y_{t-1} + \widehat{PS}_t$	-0.001 (-0.007,0.005)	0.094	-0.001 (-0.007,0.005)	0.094
(iii) $X_t + X_{t-1} + \widehat{PS}_t$	0.004 (-0.001,0.010)	0.094	-0.001 (-0.007,0.005)	0.094
(iv) $X_t + X_{t-1} + Y_{t-1} + \widehat{PS}_t$	0.000 (-0.006,0.006)	0.094	-0.001 (-0.007,0.005)	0.094
**IPW and MSMs**				
Form of $E(Y_t^{\bar{x}_t})$				
Unstabilized weights				
(i) $\omega_0 + \omega_{X1}x_t$	0.003 (-0.018,0.023)	0.332	0.087 (-0.050,0.223)	2.202
(ii) $\omega_0 + \omega_{X1}x_t + \omega_{X2}x_{t-1}$	0.007 (-0.012,0.026)	0.306	-0.015 (-0.089,0.060)	1.203
Stabilized weights				
(i) $\omega_0 + \omega_{X1}x_t$	0.000 (-0.005,0.006)	0.087	-0.060 (-0.077,-0.043)	0.275
(ii) $\omega_0 + \omega_{X1}x_t + \omega_{X2}x_{t-1}$	-0.002 (-0.008,0.005)	0.105	0.108 (-0.233,0.448)	5.495
Stabilized weights: truncated at the 1st and 99th percentiles				
(i) $\omega_0 + \omega_{X1}x_t$	0.016 (0.010,0.021)	0.084	-0.058 (-0.064,-0.053)	0.089
(ii) $\omega_0 + \omega_{X1}x_t + \omega_{X2}x_{t-1}$	0.019 (0.013,0.025)	0.099	-0.052 (-0.058,-0.045)	0.104
Stabilized weights: truncated at the 5th and 95th percentiles				
(i) $\omega_0 + \omega_{X1}x_t$	0.025 (0.020,0.030)	0.083	-0.049 (-0.054,-0.043)	0.085
(ii) $\omega_0 + \omega_{X1}x_t + \omega_{X2}x_{t-1}$	0.027 (0.021,0.033)	0.097	-0.043 (-0.049,-0.037)	0.100
Stabilized weights: truncated at the 10th and 90th percentiles				
(i) $\omega_0 + \omega_{X1}x_t$	0.043 (0.037,0.048)	0.081	-0.038 (-0.044,-0.033)	0.084
(ii) $\omega_0 + \omega_{X1}x_t + \omega_{X2}x_{t-1}$	0.045 (0.039,0.051)	0.095	-0.032 (-0.039,-0.026)	0.098
Stabilized weights: truncated at the 20th and 80th percentiles				
(i) $\omega_0 + \omega_{X1}x_t$	0.069 (0.064,0.074)	0.080	-0.025 (-0.030,-0.020)	0.081
(ii) $\omega_0 + \omega_{X1}x_t + \omega_{X2}x_{t-1}$	0.068 (0.062,0.074)	0.093	-0.021 (-0.027,-0.015)	0.096

continued on next page

continued from previous page

Model	Independence		Unstructured	
	Bias (95% CI)	SD	Bias (95% CI)	SD
Simulation scenario 3				
**Sequential conditional mean models**				
Form of $E(Y_t \| \bar{X}_t, \bar{Y}_{t-1})$				
(i) X_t	0.255 (0.250,0.260)	0.083	0.198 (0.193,0.203)	0.079
(ii) $X_t + Y_{t-1}$	0.108 (0.103,0.112)	0.075	0.050 (0.045,0.055)	0.077
(iii) $X_t + X_{t-1}$	-0.002 (-0.007,0.004)	0.089	-0.001 (-0.006,0.004)	0.086
(iv) $X_t + X_{t-1} + Y_{t-1}$	-0.002 (-0.007,0.003)	0.087	0.002 (-0.003,0.008)	0.087
**Sequential conditional mean models using propensity scores**				
Form of $E(Y_t \| \bar{X}_t, \bar{Y}_{t-1}, \widehat{PS}_t)$				
(i) $X_t + \widehat{PS}_t$	-0.001 (-0.006,0.005)	0.087	0.001 (-0.004,0.007)	0.086
(ii) $X_t + Y_{t-1} + \widehat{PS}_t$	-0.001 (-0.006,0.004)	0.087	0.006 (0.000,0.011)	0.087
(iii) $X_t + X_{t-1} + \widehat{PS}_t$	-0.002 (-0.008,0.003)	0.088	-0.001 (-0.006,0.004)	0.086
(iv) $X_t + X_{t-1} + Y_{t-1} + \widehat{PS}_t$	-0.002 (-0.007,0.003)	0.087	0.003 (-0.003,0.008)	0.087
**IPW and MSMs**				
Form of $E(Y_t^{\bar{x}_t})$				
Unstabilized weights				
(i) $\omega_0 + \omega_{X1} x_t$	0.021 (0.007,0.036)	0.236	-0.016 (-0.044,0.012)	0.454
(ii) $\omega_0 + \omega_{X1} x_t + \omega_{X2} x_{t-1}$	0.015 (0.001,0.029)	0.223	0.003 (-0.025,0.030)	0.448
Stabilized weights				
(i) $\omega_0 + \omega_{X1} x_t$	0.254 (0.249,0.259)	0.081	0.198 (0.193,0.203)	0.080
(ii) $\omega_0 + \omega_{X1} x_t + \omega_{X2} x_{t-1}$	-0.003 (-0.008,0.003)	0.087	-0.001 (-0.007,0.004)	0.086
Stabilized weights: truncated at the 1st and 99th percentiles				
(i) $\omega_0 + \omega_{X1} x_t$	0.254 (0.249,0.259)	0.081	0.197 (0.192,0.202)	0.080
(ii) $\omega_0 + \omega_{X1} x_t + \omega_{X2} x_{t-1}$	-0.002 (-0.008,0.003)	0.087	-0.001 (-0.007,0.004)	0.086
Stabilized weights: truncated at the 5th and 95th percentiles				
(i) $\omega_0 + \omega_{X1} x_t$	0.255 (0.249,0.260)	0.081	0.198 (0.193,0.203)	0.080
(ii) $\omega_0 + \omega_{X1} x_t + \omega_{X2} x_{t-1}$	-0.002 (-0.008,0.003)	0.087	-0.001 (-0.007,0.004)	0.086
Stabilized weights: truncated at the 10th and 90th percentiles				
(i) $\omega_0 + \omega_{X1} x_t$	0.255 (0.250,0.260)	0.081	0.198 (0.193,0.203)	0.080
(ii) $\omega_0 + \omega_{X1} x_t + \omega_{X2} x_{t-1}$	-0.002 (-0.008,0.003)	0.087	-0.001 (-0.007,0.004)	0.086
Stabilized weights: truncated at the 20th and 80th percentiles				
(i) $\omega_0 + \omega_{X1} x_t$	0.255 (0.250,0.260)	0.082	0.198 (0.193,0.203)	0.080
(ii) $\omega_0 + \omega_{X1} x_t + \omega_{X2} x_{t-1}$	-0.002 (-0.008,0.003)	0.088	-0.001 (-0.006,0.004)	0.086

continued on next page

continued from previous page

Model	Independence		Unstructured	
	Bias (95% CI)	SD	Bias (95% CI)	SD
Simulation scenario 4				
**Sequential conditional mean models**				
Form of $E(Y_t \| \bar{X}_t, \bar{Y}_{t-1})$				
(i) X_t	-0.002 (-0.007,0.003)	0.083	-0.001 (-0.006,0.004)	0.078
(ii) $X_t + Y_{t-1}$	-0.091 (-0.096,-0.086)	0.079	-0.001 (-0.006,0.005)	0.086
(iii) $X_t + X_{t-1}$	-0.002 (-0.007,0.004)	0.089	-0.001 (-0.006,0.004)	0.086
(iv) $X_t + X_{t-1} + Y_{t-1}$	-0.002 (-0.008,0.003)	0.087	-0.001 (-0.006,0.004)	0.085
**Sequential conditional mean models using propensity scores**				
Form of $E(Y_t \| \bar{X}_t, \bar{Y}_{t-1}, \widehat{PS}_t)$				
(i) $X_t + \widehat{PS}_t$	-0.002 (-0.008,0.003)	0.087	-0.001 (-0.006,0.004)	0.086
(ii) $X_t + Y_{t-1} + \widehat{PS}_t$	-0.003 (-0.009,0.002)	0.087	-0.001 (-0.007,0.004)	0.085
(iii) $X_t + X_{t-1} + \widehat{PS}_t$	-0.002 (-0.008,0.003)	0.088	-0.001 (-0.006,0.004)	0.086
(iv) $X_t + X_{t-1} + Y_{t-1} + \widehat{PS}_t$	-0.002 (-0.008,0.003)	0.087	-0.001 (-0.006,0.005)	0.085
**IPW and MSMs**				
Form of $E(Y_t^{\bar{x}_t})$				
Unstabilized weights				
(i) $\omega_0 + \omega_{X1} x_t$	0.014 (0.000,0.028)	0.228	0.079 (-0.034,0.192)	1.824
(ii) $\omega_0 + \omega_{X1} x_t + \omega_{X2} x_{t-1}$	0.015 (0.002,0.029)	0.223	0.053 (-0.014,0.119)	1.074
Stabilized weights				
(i) $\omega_0 + \omega_{X1} x_t$	-0.002 (-0.007,0.003)	0.081	-0.001 (-0.006,0.003)	0.078
(ii) $\omega_0 + \omega_{X1} x_t + \omega_{X2} x_{t-1}$	-0.002 (-0.008,0.003)	0.087	-0.001 (-0.007,0.004)	0.086
Stabilized weights: truncated at the 1st and 99th percentiles				
(i) $\omega_0 + \omega_{X1} x_t$	-0.002 (-0.007,0.003)	0.081	-0.001 (-0.006,0.003)	0.078
(ii) $\omega_0 + \omega_{X1} x_t + \omega_{X2} x_{t-1}$	-0.002 (-0.008,0.003)	0.087	-0.001 (-0.007,0.004)	0.086
Stabilized weights: truncated at the 5th and 95th percentiles				
(i) $\omega_0 + \omega_{X1} x_t$	-0.002 (-0.007,0.003)	0.081	-0.001 (-0.006,0.003)	0.078
(ii) $\omega_0 + \omega_{X1} x_t + \omega_{X2} x_{t-1}$	-0.002 (-0.008,0.003)	0.087	-0.001 (-0.007,0.004)	0.086
Stabilized weights: truncated at the 10th and 90th percentiles				
(i) $\omega_0 + \omega_{X1} x_t$	-0.002 (-0.007,0.003)	0.081	-0.001 (-0.006,0.003)	0.078
(ii) $\omega_0 + \omega_{X1} x_t + \omega_{X2} x_{t-1}$	-0.002 (-0.008,0.003)	0.087	-0.001 (-0.006,0.004)	0.086
Stabilized weights: truncated at the 20th and 80th percentiles				
(i) $\omega_0 + \omega_{X1} x_t$	-0.002 (-0.007,0.003)	0.081	-0.001 (-0.006,0.003)	0.078
(ii) $\omega_0 + \omega_{X1} x_t + \omega_{X2} x_{t-1}$	-0.002 (-0.008,0.003)	0.088	-0.001 (-0.006,0.004)	0.086

Web Appendix 5 Handling study drop-out and missing data in SCMMs and IPW of MSMs

Drop-out is common in longitudinal studies. SCMMs are valid without modification when there is study drop out under the assumption that there are no variables which predict drop out and are associated with model covariates but not included in the model ('ignorable drop out'). This is likely to be more realistic when the exposure is measured close to the time of the outcome. In contrast, in IPW estimation of MSMs, drop out is handled using inverse probability of censoring weights (see e.g. [4]). A further advantage of SCMMs is that estimates from this analysis are valid in the presence of missing data in time-varying covariates provided the missingness is independent of the outcome given other covariates in the model [5], whereas in IPW of MSMs missingness would have to be handled using additional weights.

References

[1] Pepe M, Anderson G. A cautionary note on inference for marginal regression models with longitudinal data and general correlated response data. *Commun. Stat. Simul. Comput.* 1994; **23**(4):939–951.

[2] Vansteelandt S. On confounding, prediction and efficiency in the analysis of longitudinal and cross-sectional clustered data. *Scandinavian Journal of Statistics* 2007; **34**(3):478–498.

[3] Chamberlain G. Comment: Sequential moment restrictions in panel data. *Journal of Business and Economic Statistics* 1992; **10**(1):20–26.

[4] Daniel R, Cousens S, De Stavola B, *et al.*. Methods for dealing with time-dependent confounding. *Statistics in Medicine* 2013; **32**(9):1584–1618.

[5] Bartlett J, Carpenter J, Tilling K, *et al.*. Improving upon the efficiency of complete case analysis when covariates are MNAR. *Biostatistics* 2014; **15**(4):719–730.
